# Answers to Querists

**Published:** 1867-07

**Authors:** 


ANSWERS TO QUERISTS. *Query* 1*st.*—Is it *ever* proper to destroy the nerve of a temporary tooth? *Answer.*—We prefer such palliative treatment as the application of creasote on a pellet of cotton to the exposed pulp; and only where such means fail to give relief, and the preservation of the tooth, for a time at least, is necessary, to avoid the consequences resulting from its premature extraction, such as irregularity of the permanent, &c., do we use devitalizing agents.

For some time past, in our own practice, we have applied carbolic acid to the exposed pulps of both permanent and temporary teeth, and have obtained satisfactory results from its use. It frequently happens in the preparation of cavities in both temporary and permanent teeth, that the decay has progressed so far that its removal necessarily exposes the pulp. In such cases as these we should endeavor to preserve the vitality of the pulp, instead of destroying it; and the agent known as Carbolic acid will in the majority of cases, prove effectual in accomplishing this result.

Carbolic Acid or Phenole, is a colorless, crystallized solid, of a taste at first pungent and then sweet, and with an odor like that of tar. It was discovered in 1834, and exists in considerable quantity in coal tar. Chemically, it is considered as an alcohol rather than an acid and is soluble in alcohol, ether and benzole: for dental use it can be rendered fluid by the addition of a little cologne water.

The manner of using this agent is as follows: remove the carious portion as thoroughly as is possible without wounding the pulp; then syringe the cavity with luke warm water, and carefully dry with cotton, which being a soft material, is preferable in these cases, to bibulous paper.

A small pellet of cotton is then saturated with carbolic acid, and introduced on the point of an excavator into the cavity, and gently pressed upon the exposed portion of the pulp; another pellet of cotton is applied over this, and the whole is allowed to remain for ten or twelve minutes.

The cotton pellets are then removed and a small piece of bibulous paper, folded two or three layers thick, and slightly moistened with the Carbolic acid, is placed at the bottom of the cavity over the exposed portion of the pulp, and in contact with it, and the operation completed by the introduction of a filling of Hill's Stopping. After remaining several months in the cavity, the Hill's Stopping filling may be removed, when, in the great majority of cases, if the proper care has been observed in the previous treatment, the nerve will be found in such a condition as to justify, in the case of a permanent tooth, the introduction of a gold filling. It is well, however, to allow the Hill's Stopping filling, to remain in the tooth until it has so worn away as to be no longer serviceable; for a properly introduced Hill's Stopping filling, until so worn by the friction of masticating, will preserve the tooth as effectually as one of any other material.

Where the pulp of a temporary tooth is not exposed, but the decay has progressed so far as to render it susceptible to the influence of changes of temperature, such palliative treatment as the application of creasote, or carbolic acid will prove effectual. In many cases the introduction of a pellet of cotton saturated with sandarach varnish, and allowed to remain one or two days will prove serviceable, by acting as a non-conductor, excluding air, and protecting the sensitive portion from the action of irritating agents.

Where, however, owing to the condition of the pulp, such palliative treatment fails, and its destruction becomes necessary, we do not hesitate in the case of temporary teeth, to use the preparation of Dr. Warren Welsh, known as *Welch's Nerve Paste*, which contains but one grain of arsenious acid in a quantity sufficient for more than two hundred applications. Applied to the pulps of temporary teeth, this preparation will prove effectual in destroying the vitality in from three to six hours, when it should be removed and the cavity well syringed with tepid water.

*Query 2nd.*—"What material is the most suitable for filling temporary teeth?" *Answer.*—We prefer Hill's Stopping to any other material for the following reasons: it is a non-conducting substance, consequently impressions of heat and cold are not conducted through it to the sensitive portions of the tooth; it is readily introduced, and but little pressure is necessary to properly condense it; in case of subsequent trouble a filling of this material can be readily removed.

Some advocate the use of Amalgam, others Os-Artificiel as materials for filling temporary teeth. In the use of Amalgam we have a material which readily conducts impressions of heat and cold, to say nothing of the effects resulting from the absorption of the mercury by the circulation; we also have in Amalgam a material so hard that it is impossible, in many cases, to remove it, should trouble occur, without subjecting the patient to great torture, and in the majority of such cases the tooth would have to be sacrificed. Os-Artficiel is less objectionable than Amalgam; the greatest trouble in the use of this material being the length of time it is necessary to protect it from saliva, after it is introduced, in order that it may properly harden.

*Query 3d.*—"Is it necessary to lance the gums for the extraction of temporary teeth?" *Answer.*—We regard this operation as altogether unnecessary in the case of temporary teeth.

The connection of these teeth with the soft structures is slight, and the operation of lancing the gums has, in the majority of cases, such a terrifying effect upon the patients, that they will not submit to the application of the forceps.

Where the beaks of the forceps are carefully applied to the necks of the teeth, and no part of the gum is inclosed between them, laceration will not occur.

Want of space prevents us from answering other Queries in this number.

---

